# Chicago Medical Society; Meeting November 1

**Published:** 1886-12

**Authors:** 


### SOCIETY REPORTS.

CHICAGO MEDICAL SOCIETY.

*Stated Meeting, November 1st, 1886.*—E. J. DOERING, M.D., PRESIDENT, in the chair.

PROFESSOR ALBERT E. HOADLEY read a report of five cases of stricture of the rectum, with remarks on the treatment of the more severe forms, which appears in full in another part of the MEDICAL JOURNAL AND EXAMINER.

DISCUSSION.

DR. J. FRANK thought that if the author had divided his paper into relief for malignant strictures, and treatment for non-malignant strictures, it would have been a better classification. He had not had much experience with malignant strictures, but had divided one in the manner described by the author, by cutting down through the cellular tissue. He thought there was little danger in performing the operation, and was surprised at the small amount of hæmorrhage. But the benefit from the operation lasted only for a short time; there was relief at first, but in six or eight weeks the same symptoms returned. Even in extirpated cancerous growths, as far as his information went, they generally return within a year. He had had one case in which the whole cancerous growth was extirpated, but in six or eight months it commenced to return and in a year's time the patient died with cancer. He thought that in dividing the strictures care must be taken not to go too far up the bowel, or too deep, as the peritoneum might be cut into.

PROFESSOR HOADLEY, in closing the discussion, said: Dr. Frank suggests that we be careful in dividing the strictures high up; if we do not divide them higher than we can reach with the finger, dividing them in the posterior line, there is little danger of opening the peritoneal cavity. The peritoneum does not come down, as a rule, where it can be reached with the finger. In regard to hæmorrhage, those cases sometimes bleed profusely even though they are divided right in the median line, where we least expect to find blood vessels; the tissues become vascular, new vessels form, and it is necessary to tampon the wound, and in doing so it is best to put in a tube to relieve the bowels of gas. The suggestion made in reference to the division of the paper is quite proper; perhaps it would have been better to have said, report of cases illustrating a treatment. The treatment is palliative in malignant strictures, and in non-malignant strictures sometimes effects a cure. In answer to the question, "How many inches up may we go?" it is a rather difficult matter to reach the peritoneum of the posterior wall of the rectum with the finger, even if the sphincter is divided; one can reach about four inches with the finger by pushing hard; it would be unsafe to divide a stricture further than one could reach with his finger. Even the inferior mesenteric artery (superior hæmorrhoidal) comes down sometimes, before it bifurcates into the lateral branches, within reach of the finger, and may be divided, but that is no drawback to the operation, because one can put a tampon into the rectum so firmly that all hæmorrhage can be perfectly controlled, and on the next day one may remove about half of the tampon to relieve tension, and the remaining half will tumble out itself three or four days later. It has been his experience as well as that of others that the vessels there can be controlled with the tampon very securely and with perfect safety. It is best to prepare for it and always tampon where one makes that division, because moderate hæmorrhage is sometimes quite persistent.

DR. A. J. OCHSNER read a report of a case of actinomycosis, which appears elsewhere in this number of the JOURNAL AND EXAMINER.

DISCUSSION.

PROFESSOR R. H. BABCOCK said, the case is one of exceeding interest from its rarity, and particularly as it is a case occurring in this country, and one of very few that have been recorded, and in this case the diagnosis is so unquestionable that the interest is all the greater. We know that in cattle the disease is manifested by tumefaction in various organs, whereas in human beings it is by suppuration and metastatic abscesses. The disease in the human being may affect any of the organs, not merely the lungs, but particularly the viscera of the abdomen. He inquired of Dr. Ochsner if there are signs of the disease having attacked other parts of the body than those mentioned, any symptoms which lead him to infer that the digestive organs or the stomach are infected.

DR. R. TILLEY said, this is certainly one of the most interesting questions that has been brought before the society for a considerable time. In the case presented to-night the disease seems to have originated in the antrum, and is therefore especially interesting to those engaged in treating affections of the nose and throat, and of the teeth. As in the history of this case there was a considerable amount of pain associated with the eyes, it is of interest to the ophthalmologist, and as it is now associated with the lungs, it is of interest to those engaged in the study of affections of the lungs. As this case, together with the last case presented to the society, in all probability constitute the only indisputable cases that have appeared in English literature, he believed it would be of sufficient interest to the society to ask Dr. Ochsner to carry the investigation still further and try to produce the disease by inoculation on one of the lower animals. He would move, at the proper time, that the society place at Dr. Ochsner's disposal the necessary funds, and he would suggest that Dr. Ochsner accompany his report with a diagram of the fungus as it appears under the microscope. He had looked at the various schematic sketches that are published, and he claims that it would be absolutely impossible for anyone with only the information afforded in these articles, without further study, to diagnosticate the fungi as they appear in the specimens presented. In Dr. Belfield's book, the diagram is more in correspondence with those we see to-night, but those that are in Councilman's article in Wood's Reference Handbook certainly do not present such an appearance.

DR. J. D. SKEER inquired as to the condition of the lung at the present time, whether cavernous or indurated.

DR. FRANK BILLINGS endorsed the remarks of Dr. Tilley, and thought the society should afford Dr. Ochsner means to carry on the investigation. It is not quite settled how this fungus is carried from one tissue to another; in one case it has been proven that it was carried to the heart by ulceration into one of the jugular veins, and it is known that it will spread through contiguous tissue as through the diaphragm from the pleural cavity to the peritoneal cavity. A pure cultivation of the fungus has been made at Berlin.

DR. HAROLD N. MOYER said there is great uncertainty as to the manner in which the fungus obtains access to the tissues.

He would ask Dr. Ochsner if there is anything in the history of this case that would clear up that point. Two very interesting cases have been recently reported, in one case lung actinomycosis was diagnosticated during life, and after death of the patient a large actinomycotic mass was found in the lower portion of the upper lobe of the left lung, and in the center of the mass a piece of tooth was found. Israel (Center. Bl. f. d. Med. Wesseusch) demonstrated actinomycosis in the sputum of that case, and so far as he knew it is the only case on record in which a diagnosis was made from the sputum. The other case bears on the manner in which the fungus obtains access to the tissues and is reported by Soltmann (Jahrb. f. Kinderhkde). A child while at play swallowed a head of what is known as fox-grass, which was followed by severe pain, difficulty in swallowing, and difficulty in respiration. In a few weeks the head of grass was discharged in an abscess at the left of the vertebral column. In less than six months this case developed actinomycosis. The theory of Israel regarding the first case is that the parasite was carried by the piece of tooth directly into the lung. In the second case the fox grass undoubtedly carried it directly into the mediastinum, which was followed by secondary involvement of the tissue of the lung.

PROFESSOR W. T. BELFIELD said, he would like to say in reference to Dr. Tilley's proposition, that experiments on the cultivation or transmission of this fungus ought to be made from fungi obtained from animals and not from man; if from the latter, from pus taken from abscess cavities around the jaws. The reason is that fungi obtained in the sputum are far more delicate in appearance and for preservation. He has found that after a few days they disappear, or at least are extremely hard to find, and it is stated by those who have had a great deal of experience that the fungi obtained from the sputum are not so robust as those obtained from cattle or from pus-cavities, aside from the air passages, in man. In justice to Dr. Shirmer, he would say that in the case reported by him, the fungus was recognized in the sputum and a diagnosis of lung-actinomycosis was made.

DR. R. TILLEY said, the question with him was not that of producing the best possible sample of the fungus, but to place cases in question beyond dispute and give the society further opportunities of studying the question. Necessarily American physicians must be ignorant of the question as a whole, because it is impossible to get that accurate information from literature that we can get from the study of the manifestations of the disease. He would not discriminate between this case and that of Dr. Schirmer; on the contrary, if there is a probability of developing the fungus from pus, and an animal is chosen, he did not think there would be any objection to taking samples from both cases to inoculate the same animal.

DR. OCHSNER in closing the discussion said, as far as we know at present there is no evidence of the existence of actinomycosis in any other portion of the body; the probable reason of this is the fact that the patient has been exceedingly careful never to swallow any of the discharge from the abscess or any of the sputum. The accumulation of the fungus in the lungs is probably not sufficient for ulceration to have taken place into any of the other organs. The amount of sputum during twenty-four hours is between one and two ounces, and some days there will be one or two of these accumulations of actinomyces of considerable size and a few very small points, and on some days it is impossible to demonstrate actinomycosis. On Saturday there were a dozen quite large accumulations and quite a number of small ones; so that judging from the number we find in the sputum, and from the physical signs, which are a decrease in the motion of the left side, mucous râles, and very slight dullness on the left side above the fifth rib, the accumulations are not very great, and we do not find any signs of the existence of actinomyces in any other organ, which answers also Dr. Skeer's question concerning the condition of the lungs at present. Concerning the access to the tissue, the patient had teeth removed from the left side of the upper jaw a number of times, during which time he was constantly handling animals suffering from lumpy-jaw, which is the same as actinomycosis in man, and constantly handling objects that came in contact with these animals. Another point of interest which might give us some light as to the possible introduction of actinomycosis in man is this: The patient told him that on the farm whenever one animal has lumpy-jaw, a number of animals are very likely to have it soon after. This happens generally in the spring of the year, or late in winter, when animals are likely to rub their necks on the fences. He thinks these animals with lumpy-jaw rubbing their necks on the fences are likely to leave some of the actinomyces and the healthy animals are likely to scratch their skin and break the hide, and in that way become infected. The plants are exceedingly small and could with great ease be introduced into the mouth and the cavities of the teeth, or into the jaw from which teeth had been extracted. Drs. Ross and Robison are treating the patient at present by means of the pneumatic cabinet, introducing into the lungs 1-1000 solution of bi-chloride of mercury. It seems that more of the actinomyces have been coughed up since this treatment began, but it has been of such short duration that nothing certain can yet be said.

THE COMMITTEE ON PATHOLOGY

reported through Professor W. T. Belfield the examination of the case of actinomycosis hominis presented to the society on September 6th. The patient is a man about twenty-five years old, emaciated and feeble. The lateral diameter of the neck is considerably increased by inflammatory thickening of the superficial cervical tissues, which are hard and unyielding; on either side of the neck is a jagged scar and small fistulous opening from which issues a slight serous discharge. The jaws could be separated to only a slight extent; but so far as could be determined, the mouth and throat presented nothing abnormal; no carious teeth were detected.

Dullness on percussion and broncho-vesicular breathing were found over the apex of each lung; on the left side in the supra-clavicular region and first intercostal space anteriorly; on the right side down to the third rib.

The patient coughs frequently and occasionally raises considerable sputum. About an ounce of sputum was collected from which slides were prepared and several specimens of actinomyces were detected.

On September 28th an incision was made in the left side of the neck, giving exit to a small quantity of pus, containing actinomyces. It was the opinion of Professor Belfield that this patient is the subject of actinomycosis.

Professor Belfield said that he had been informally requested by the President to examine the case presented by

Dr. Ochsner, and had found the patient exactly as described. He thought there was no question about the genuineness of the specimens exhibited. The diagnosis rested altogether on the detection of the fungi in the sputum, because if that were absent the physical signs might be due to some other cause, but he thought the physical signs in this case were caused by the fungi in the lungs.

On motion of Dr. Tilley the sum of fifty dollars was appropriated for the purpose of making a series of experiments with actinomyces.

